# Safety and efficacy of daclizumab in relapsing-remitting multiple sclerosis: 3-year results from the SELECTED open-label extension study

**DOI:** 10.1186/s12883-016-0635-y

**Published:** 2016-07-26

**Authors:** Ralf Gold, Ernst-Wilhelm Radue, Gavin Giovannoni, Krzysztof Selmaj, Eva Havrdova, Dusan Stefoski, Till Sprenger, Xavier Montalban, Stanley Cohan, Kimberly Umans, Steven J. Greenberg, Gulden Ozen, Jacob Elkins

**Affiliations:** 1 Department of Neurology, St. Josef-Hospital/Ruhr-University Bochum, Bochum, Germany; 2 Medical Image Analysis Center, University Hospital Basel, Basel, Switzerland; 3 Queen Mary University of London, Blizard Institute, London School of Medicine and Dentistry, London, UK; 4 Medical University of Lodz, Lodz, Poland; 5 First Faculty of Medicine, Charles University in Prague, Prague, Czech Republic; 6 Rush University Medical Center, Chicago, IL USA; 7 DKD Helios Klinik Wiesbaden, Wiesbaden, Germany; 8 Hospital Vall d’Hebron University, Barcelona, Spain; 9 Providence Multiple Sclerosis Center, Portland, OR USA; 10 Biogen, Cambridge, MA USA; 11 AbbVie Biotherapeutics Inc., Redwood, CA USA

**Keywords:** Relapsing-remitting multiple sclerosis, Daclizumab, Safety, Efficacy

## Abstract

**Background:**

Daclizumab is a humanized monoclonal antibody against CD25 that modulates interleukin 2 signaling. The SELECT TRILOGY of clinical studies (SELECT/SELECTION/SELECTED) evaluated the safety and efficacy of daclizumab in patients with relapsing-remitting multiple sclerosis (RRMS). We report the long-term safety and efficacy of daclizumab 150 mg subcutaneous every 4 weeks in patients with RRMS in the SELECTED open-label extension study.

**Methods:**

An interim intent-to-treat analysis of all enrolled patients was performed in January 2014 for this ongoing study.

**Results:**

The SELECTED study enrolled 90 % of patients who completed SELECTION. In the safety and efficacy analysis (*N* = 410), median treatment time in SELECTED was 25 months (range, <1–45). Adverse events (AEs) were reported in 76 % of patients, serious AEs (SAEs) excluding MS relapse in 16 %, and treatment discontinuation due to AEs including multiple sclerosis (MS) relapse in 12 %. AEs were primarily of mild to moderate severity, and common AEs (≥10 %), excluding MS relapse, were nasopharyngitis (12 %) and upper respiratory tract infection (12 %). Most commonly reported SAEs (in ≥3 patients), excluding MS relapses, were increased serum hepatic enzymes, pneumonia, ulcerative colitis, and urinary tract infection (<1 % each). Incidences of AE groups of interest include cutaneous events (28 %), cutaneous SAEs (2 %), gastrointestinal SAEs (2 %), hepatic SAEs, (1 %) and malignancies (1 %). The incidence of AEs, SAEs, and treatment-related study discontinuations did not increase over time and no deaths were reported. The adjusted annualized relapse rate (95 % confidence interval (CI)) analyzed at 6-month intervals was 0.15 (0.10–0.22) for weeks 97–120 and 0.15 (0.10–0.21) for weeks 121–144. In year 3, the adjusted mean (95 % CI) number of new/newly enlarging T2 hyperintense lesions was 1.26 (0.93–1.72) and the mean (median) annualized change in brain volume was −0.32 % (−0.34 %).

**Conclusions:**

The AE incidence did not increase with extension of therapy into year 3 in SELECTED; the safety profile was similar to that previously observed. The clinical efficacy of daclizumab was sustained over the 3 years comprising the SELECT TRILOGY, although potential selection bias cannot be excluded.

**Trial registration:**

Clinicaltrials.gov NCT01051349; first registered January 15, 2010.

**Electronic supplementary material:**

The online version of this article (doi:10.1186/s12883-016-0635-y) contains supplementary material, which is available to authorized users.

## Background

Effective disease activity control in patients with relapsing-remitting multiple sclerosis (RRMS) is critical for improved long-term outcomes and requires long-term treatment. Hence, it also is essential to establish the long-term safety and efficacy of multiple sclerosis (MS) disease-modifying therapies (DMTs).

Interleukin 2 (IL-2) signaling has a central role in both immune system activation and regulation [1]. IL-2 receptors are expressed on a variety of immune cells, including CD4^+^ and CD8^+^ T cells, regulatory T cells, CD56^bright^ natural killer cells, and myeloid dendritic cells [2]. IL-2 signaling supports immune system activation through CD4^+^ and CD8^+^ T cell cytokine secretion, T effector cell expansion, and CD8^+^ T cell and CD56^bright^ natural killer cell cytotoxicity, while also supporting immune system regulation by promoting regulatory T cell expansion and survival [2].

Daclizumab high-yield process (daclizumab)^1^ is a humanized monoclonal antibody that modulates IL-2 signaling by binding to the IL-2 receptor alpha chain (CD25), thereby inhibiting assembly of the high-affinity IL-2 receptor (CD25/CD122/CD132) and shifting IL-2 signaling to cells that express the intermediate-affinity IL-2 receptor (CD122/CD132) [2]. This transient shift antagonizes proinflammatory activated T cells and leads to the expansion of immunoregulatory CD56^bright^ natural killer cells [2]. Daclizumab is hypothesized to inhibit disease activity and slow disease progression by enhancing endogenous mechanisms of immune tolerance through expansion of immunoregulatory CD56^bright^ natural killer cells and reducing early T cell activation [2].

The SELECT TRILOGY (SELECT [3], SELECTION [4], SELECTED) of clinical studies was designed to evaluate the efficacy and safety of daclizumab in patients with RRMS. SELECT was a multicenter, randomized, double-blind, phase 2 study that evaluated the efficacy and safety of daclizumab 150 and 300 mg subcutaneous (SC) every 4 weeks for 1 year versus placebo [3]. In SELECTION, a 1-year double-blind extension of SELECT, placebo-treated patients were randomized to daclizumab 150 or 300 mg SC, and daclizumab-treated patients either continued their previous dosage of daclizumab or underwent a 24-week washout period followed by re-initiation of daclizumab at their previous dose [4]. SELECTED is an ongoing, open-label, extension study of SELECTION that is being conducted to assess the long-term safety and efficacy of daclizumab monotherapy (150 mg SC every 4 weeks).

In SELECT, daclizumab 150 mg reduced adjusted annualized relapse rate (ARR), disability progression, and magnetic resonance imaging (MRI) lesion activity compared with placebo [3]. Infections, cutaneous adverse events (AEs), and hepatic enzyme elevations were more common with daclizumab compared with placebo in SELECT [3]. In SELECTION, the incidence of AEs was similar to that observed in SELECT and efficacy was maintained in the second year among patients receiving continuous daclizumab treatment [4]. This paper reports interim safety and efficacy data for an international cohort of patients with RRMS treated with 150 mg daclizumab in SELECTED.

## Methods

### Study design

**Fig. 1:**
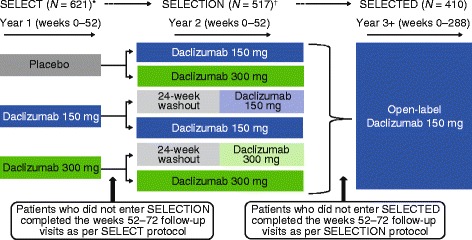
SELECT TRILOGY study design. The dosing regimen for study drug (ie, placebo or daclizumab) was subcutaneous (SC) every 4 weeks in all three studies of the SELECT TRILOGY [3, 4]. *Gold et al. [3]. ^†^Giovannoni et al. [4]

SELECTED is an ongoing, single-arm, open-label extension study to evaluate the long-term safety and efficacy of daclizumab 150 mg SC every 4 weeks for up to 6.5 years from enrollment in patients with RRMS who completed SELECT and SELECTION (Fig. 1). The study is being conducted at 66 investigational sites in eight countries: the Czech Republic, Germany, Hungary, India, Poland, Russia, Ukraine, and the United Kingdom. The first patient was enrolled and treated on 31 March 2010; enrollment is now completed and the study is ongoing. The primary objective of SELECTED is to assess the safety of extended treatment with daclizumab monotherapy in patients with RRMS; efficacy is being assessed as a secondary objective.

Patient eligibility was determined at week 52 of SELECTION, and this visit also served as the baseline visit of SELECTED. At enrollment in SELECTED, patients had previously received 1–2 years of treatment with daclizumab 150 or 300 mg SC (with or without a washout with total duration of 24 weeks comprised of the last 4 weeks of SELECT and the 20 weeks of SELECTION). All eligible patients received daclizumab 150 mg SC every 4 weeks, and had clinic visits scheduled every 4 weeks for the first 12 weeks in the study, followed by clinic visits scheduled every 12 weeks for up to 6 years of continuous treatment.

### Patients

To be included in the study, patients must have completed 52 weeks of both SELECT and SELECTION, been compliant with the SELECTION protocol, provided informed consent for SELECTED, and met other general eligibility criteria. Women of childbearing potential must have agreed to practice effective contraception during the study and for 4 months after their last dose of study treatment. Key exclusion criteria included: a significant change in medical status from a previous study that precluded administration of daclizumab, permanent discontinuation of study treatment in SELECTION due to an AE, enrollment in any other investigational drug study, or ongoing treatment with any approved or experimental DMT for MS. Eligibility criteria for SELECT and SELECTION have been reported previously [3, 4].

### Safety assessment

Safety and tolerability assessments included AE monitoring, physical and neurological exams, vital signs, electrocardiograms, and clinical lab evaluations (hematology, blood chemistry, liver function panel, and urinalysis). For each AE, investigators rated its severity based on guidance in the protocol and determined whether it also met the regulatory criteria for a serious AE (SAE). Patients who experienced a clinically significant cutaneous AE (defined as rash, dermatitis, eczema, acne, or folliculitis) were referred to a dermatologist. Liver function testing (including alanine transaminase [ALT], aspartate transaminase [AST], and total bilirubin) was performed monthly.

### Efficacy assessment

Relapses were defined as new or recurrent neurological symptoms, not associated with fever or infection, lasting at least 24 h, and accompanied by new objective neurological findings upon examination by the neurologist. New or recurrent neurological symptoms that evolved gradually over months were considered disability progression, not an acute relapse. New or recurrent neurological symptoms that occurred less than 30 days following the onset of a protocol-defined relapse were considered part of the same relapse. Brain MRI scans were performed annually and read for efficacy outcomes at a central reading institution (Medical Image Analysis Center, Basel, Switzerland).

### Statistical analysis

For both safety and efficacy parameters, changes from baseline were evaluated based on the first dose of daclizumab in either SELECT (patients randomized to daclizumab in SELECT) or SELECTION (patients randomized to placebo in SELECT and daclizumab in SELECTION; Fig. 1).

#### Safety

The safety analysis was performed on all patients who received at least one dose of daclizumab in SELECTED. All treatment-emergent AEs during SELECTED were included in the evaluation of safety. Treatment-emergent AEs included any event that either occurred or worsened in severity after the first dose of study treatment in SELECTED up to 180 days after the last dose of daclizumab. AEs were coded using the Medical Dictionary for Regulatory Activities (MedDRA) Version 16.1. Hepatic events were identified with the Standardized MedDRA Query (SMQ) “drug related hepatic disorders.”

#### Efficacy

The intent-to-treat efficacy analysis was performed on SELECTED patients starting from the first dose of daclizumab treatment in patients randomized to daclizumab in SELECT or first dose in SELECTION for patients randomized to placebo in SELECT and daclizumab in SELECTION. The ARR was calculated by tabulating the total number of relapses experienced divided by the total number of patient-years. The adjusted ARR was estimated from a Poisson regression adjusted for the number of relapses in the year before study entry. Relapse rates were also estimated by time interval from the first dose of daclizumab received (0–24, 25–48, 49–72, 73–96, 97–120, 121–144 weeks). The adjusted mean number of T2 hyperintense lesions during years 1, 2, and 3 of daclizumab treatment was estimated from a negative binomial regression adjusted for baseline number of T2 hyperintense lesions. The annualized percentage brain volume change (PBVC) was determined with Structural Image Evaluation using Normalization of Atrophy (SIENA) and was calculated as percentage change divided by the number of days since the last scan, multiplied by 365.25.

This report adheres to CONSORT guidelines.

## Results

### Patients

**Fig. 2:**
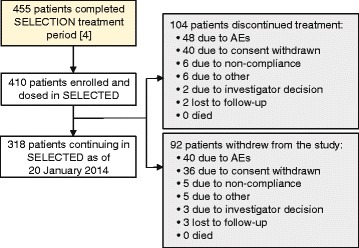
SELECTED trial profile. Since the duration of SELECTED is over 5 years, based on first patient dosed date of 31 March 2010, no patient had completed the study as of the data cutoff for this manuscript. *AE* Adverse event

Of all patients who completed SELECTION, 410 (90 %) enrolled and were dosed with daclizumab in SELECTED (Fig. 2). The safety and efficacy populations for this interim analysis were the same, comprising all patients who were dosed in SELECTED. SELECTED baseline patient characteristics are shown in Table 1. At the time of this interim analysis, study enrollment was complete, and all 410 enrolled patients had received at least one dose of daclizumab in SELECTED. The median time on daclizumab treatment in SELECTED was 25 (range, <1–45) months (854 patient-years). Across the SELECT TRILOGY, patients in SELECTED had received a median of 48 (range, 13–74) doses of daclizumab. At the time of the interim analysis, 296 (72 %) patients had received more than 40 total doses and 168 (41 %) had received more than 50 total doses. During the study period covered by the interim analysis, 104 (25 %) patients discontinued treatment and 92 (22 %) patients withdrew from the study (Fig. 2).

**Table 1:** Patient demographics and clinical characteristics at SELECTED baseline

Characteristic	Study population
	(*N* = 410)
Age, years, mean (SD)	38 (9)
Female, %	62
No. of relapses in prior study, mean (SD)^a^	0.2 (0.5)
Range	0–3
EDSS score, mean (SD)	2.7 (1.3)
Range	0–6
No. of Gd^+^ lesions, mean (SD)	0.2 (1.0)
Range	0–12
Patients with ≥1 Gd^+^ lesion(s), n (%)	40 (10)
No. of T2 hyperintense lesions, mean (SD)	46.3 (36.5)
Range	0–194
T2 hyperintense lesion volume, mm^3^, median	3868
T1 hypointense lesion volume, mm^3^, median	751
Months on treatment, median^b^	25
Range	<1–45
Doses, mean (SD)	27.7 (10.8)
Median	28.0
Range	1–49

*EDSS* Expanded Disability Status Scale, *Gd* ^+^ Gadolinium-enhancing, *SC* Subcutaneous, *SD* Standard deviation

^a^Includes all relapses in SELECTION, whether in the treatment period, randomized washout phase, or follow-up period, either confirmed or not confirmed by an independent neurology evaluation committee

^b^Time on treatment in days was derived as (date of last dose) – (date of first dose in SELECTED) + 1

### Safety overview and incidence of AEs and SAEs

AEs were summarized by three time periods and overall. The yearly incidence of AEs, SAEs, and AEs leading to discontinuation did not increase over time and no deaths were reported (Table 2). Forty-eight (12 %) patients discontinued treatment due to AEs (Table 3). Common AEs that occurred in 10 % of patients or more were MS relapse (22 %), nasopharyngitis (12 %), and upper respiratory tract infection (12 %; Table 4). The most frequently reported SAEs excluding MS relapse, were hepatic enzyme elevations, pneumonia, ulcerative colitis, and urinary tract infection (each in three patients [each less than 1 %]; Table 5).

**Table 2:** Summary of AEs in SELECTED by time intervals and overall

AE, *n* (%)	Daclizumab 150 mg SC			
	Weeks 1–48^a^	Weeks 49–96^b^	Week 97 and above^c^	Overall
	(*n* = 410)	(*n* = 387)	(*n* = 279)	(*N* = 410)
All AEs	245 (60)	222 (57)	126 (45)	312 (76)
AEs by severity^d^				
Mild	122 (30)	96 (25)	45 (16)	101 (25)
Moderate	110 (27)	115 (30)	70 (25)	178 (43)
Severe	13 (3)	11 (3)	11 (4)	33 (8)
All SAEs	53 (13)	47 (12)	34 (12)	105 (26)
SAEs (excluding MS relapse)	23 (6)	26 (7)	20 (7)	66 (16)
AEs leading to treatment discontinuation	22 (5)	17 (4)	9 (3)	48 (12)
Death	0	0	0	0

*AE* Adverse event, *MS* Multiple sclerosis, *SAE* Serious adverse event, *SC* Subcutaneous

^a^Weeks 1–48 of SELECTED represents the second year of daclizumab treatment in patients who were newly treated with daclizumab in SELECTION [4] and the third year of treatment in patients originally treated with daclizumab in SELECT [3]

^b^Weeks 49–96 represents the third year of daclizumab treatment in patients who were newly treated with daclizumab in SELECTION [4] and the fourth year of treatment in patients originally treated with daclizumab in SELECT [3]

^c^Week 97 and above represents the fourth year and above of daclizumab treatment in patients who were newly treated with daclizumab in SELECTION [4] and the fifth year and above of treatment in patients originally treated with daclizumab in SELECT [3]

^d^Patients counted in the category of maximum severity experienced in the time interval

**Table 3:** AEs leading to treatment discontinuation in three or more patients

AE, *n* (%)	Daclizumab 150 mg SC
	(*N* = 410)
Any AE leading to treatment discontinuation	48 (12)
Investigations	19 (5)
Increased ALT	11 (3)
Increased AST	5 (1)
Increased hepatic enzyme	4 (<1)
Skin and subcutaneous tissue disorders	12 (3)
Allergic dermatitis	3 (<1)
Gastrointestinal disorders	4 (<1)
Colitis	3 (<1)
Hepatobiliary disorders	4 (<1)
Infections and infestations	4 (<1)

*AE* Adverse event, *ALT* Alanine transaminase, *AST* Aspartate transaminase, *SC* Subcutaneous

**Table 4:** Common AEs (occurring in 5 % of patients or more)^a^

AE, *n* (%)	Daclizumab 150 mg SC
	(*N* = 410)
MS relapse	89 (22)
Nasopharyngitis	51 (12)
Upper respiratory tract infection	49 (12)
Increased ALT	37 (9)
Pharyngitis	35 (9)
Headache	33 (8)
Urinary tract infection	31 (8)
Back pain	29 (7)
Increased AST	28 (7)
Rash	27 (7)
Diarrhea	22 (5)
Allergic dermatitis	21 (5)
Viral respiratory tract infection	20 (5)
Bronchitis	19 (5)
Oral herpes	19 (5)

*AE* Adverse event, *ALT* Alanine aminotransferase, *AST* Aspartate aminotransferase, *MS* Multiple sclerosis, *SC* Subcutaneous

^a^AEs in ≥5 % of patients by Medical Dictionary for Regulatory Activities Preferred Term

**Table 5:** SAEs occurring in two or more patients^a^

SAE, *n* (%)	Daclizumab 150 mg SC
	(*N* = 410)
Any SAE	105 (26)
MS relapse	47 (11)
Increased hepatic enzyme	3 (<1)
Pneumonia	3 (<1)
Ulcerative colitis	3 (<1)
Urinary tract infection	3 (<1)
Bronchitis	2 (<1)
Intervertebral disc disorder	2 (<1)
Lower limb fracture	2 (<1)
Lymphadenopathy	2 (<1)
Urticaria^b^	2 (<1)

*MS* Multiple sclerosis, *SAE* Serious adverse event, *SC* Subcutaneous

^a^SAEs in two or more patients by Medical Dictionary for Regulatory Activities Preferred Term

^b^Other cutaneous SAEs are presented in Table 6

#### Infections

Infections were reported in 50 % of patients, with serious infections reported in 3 %. The incidence of infections did not increase over time, reported in 34 % of patients during weeks 1–48, 30 % during weeks 49–96, and 24 % during weeks 97 and above. The majority of infections were mild or moderate in severity. Less than 1 % of patients discontinued treatment due to infections. The common AEs of infections (occurring in 10 % or more of patients) were nasopharyngitis and upper respiratory tract infections. Serious infections occurring in two or more patients were pneumonia, urinary tract infection, and bronchitis (Table 6). There were two reports of potential opportunistic infections; one non-serious case of vulvovaginal candidiasis, which was treated with clotrimazole cream and resolved in 1 week, and one case of pulmonary tuberculosis, which occurred after receiving daclizumab for 2.5 years (33 total doses) in a Ukrainian patient where tuberculosis is endemic [5]; treatment was discontinued for this patient and the patient withdrew from the study. Daclizumab was temporarily interrupted in two patients due to serious infections; diverticulitis and bacterial pneumonia (one patient each), the latter was treated with clavulanate/amoxicillin, clarithromycin, and levofloxacin. In both cases, the events resolved and study treatment was resumed.

**Table 6:** Summary of serious infections, serious cutaneous AEs, serious hepatic AEs, and hepatic laboratory abnormalities

*n* (%)	Daclizumab 150 mg SC
Any serious infection^a^ (*n* = 410)	13 (3)
Pneumonia	3 (<1)
Urinary tract infection	3 (<1)
Bronchitis	2 (<1)
*Clostridium difficile* colitis	1 (<1)
Diverticulitis	1 (<1)
Gastrointestinal infection	1 (<1)
Hepatitis C	1 (<1)
Infectious mononucleosis	1 (<1)
Upper respiratory tract infection	1 (<1)
Any serious cutaneous AE^b^ (*n* = 410)	8 (2)
Urticaria	2 (<1)
Allergic dermatitis	1 (<1)
Erythrodermic psoriasis	1 (<1)
Photodermatitis	1 (<1)
Psoriasis	1 (<1)
Stevens-Johnson syndrome^c^	1 (<1)
Toxic skin eruption	1 (<1)
Any serious hepatic AE^d^ (*n* = 410)	5 (1)
Hepatic enzyme increased	3 (<1)
Autoimmune hepatitis	1 (<1)
Gamma-glutamyltransferase increased	1 (<1)
Hepatic laboratory abnormalities (*n* = 409)	
ALT or AST	
≥3 × ULN	37 (9)
>5 × ULN	18 (4)
>10 × ULN	11 (3)
Elevation in ALT or AST ≥3 × ULN with concurrent total bilirubin >2 × ULN	2 (<1)^e^

*AE* Adverse event, *ALT* Alanine aminotransferase, *AST* aspartate aminotransferase, *SC* Subcutaneous, *ULN* Upper limit of normal

Patients counted once at each level of summarization

^a^Serious infections defined as SAEs in the Medical Dictionary for Regulatory Activities (MedDRA) System Organ Class (SOC) Infections and Infestations

^b^Serious cutaneous AEs defined as SAEs in the MedDRA SOC Skin and Subcutaneous Tissues Disorders

^c^One case was reported as Stevens-Johnson syndrome but the diagnosis was not supported by the case details per the central independent dermatologist and the local site dermatologist (see text for details)

^d^Serious hepatic AEs defined as SAEs under the Standarized MedDRA Query (SMQ) of drug-related hepatic disorders

^e^No patients had concurrent elevations of ALT or AST ≥3 × ULN and total bilirubin >2 × ULN in the clinical database at the time of the interim analysis; however, two patients experienced such abnormalities either while hospitalized or after the data cutoff. In both cases, other factors as reported in the text that could have contributed to the events were noted

### Cutaneous AEs

Cutaneous events were reported in 28 % of patients, with serious cutaneous AEs in 2 %. The only cutaneous SAE reported in more than one patient was urticaria (in two patients; Table 6). One serious cutaneous AE was reported as Stevens-Johnson syndrome by the treating neurologist, but the diagnosis was not supported by the case details per the central independent dermatologist and the local site dermatologist assessments. The case did not meet the standard diagnostic criteria for Stevens-Johnson syndrome, as it was moderate in intensity, localized, lacked any bullous or necrotic skin lesions, and had no areas of loss, including full-thickness of the epidermis.

The most common cutaneous AEs were rash (7 %), allergic dermatitis (5 %), and eczema (3 %); the yearly incidence did not increase over time. The majority of patients experienced cutaneous AEs that were mild or moderate in severity; four (less than 1 %) patients experienced severe cutaneous AEs. Cutaneous events led to discontinuation of study treatment in 3 % of patients.

### Hepatic AEs

Adverse events of drug-related hepatic disorders, per SMQ, were reported in 15 % of the patients with SAEs reported in 1 % (Table 6). Overall, the incidence of ALT or AST elevations ≥3 × upper limit of normal (ULN) was 9 % and ALT or AST elevations >5 × ULN was 4 %. Two patients had liver transaminases elevations >3 × ULN with concurrent elevation of bilirubin values >2 × ULN. One of these patients experienced toxic liver disease considered to be secondary to treatment with valproate approximately 2.5 months after discontinuing study treatment. The second patient experienced jaundice with elevated liver function tests approximately 8 weeks after study treatment discontinuation following treatment of a skin event with herbal supplements and the use of influenza medication containing paracetamol.

### Gastrointestinal AEs

Gastrointestinal AEs, defined as AEs in the MedDRA System Organ Class of Gastrointestinal Disorders, were reported in 16 % of patients, with the majority of patients experiencing events that were mild or moderate in severity. The incidence of serious gastrointestinal AEs was 2 %. The incidence of gastrointestinal events that led to discontinuation of study treatment was less than 1 %. Six (1 %) patients reported serious inflammatory gastrointestinal events, including three cases of ulcerative colitis and one case each of colitis, Crohn’s disease, and hemorrhagic enterocolitis. Treatment included discontinuation of study treatment and standard therapies for colitis, including mesalazine, sulfasalazine, corticosteroids, and azathioprine. The majority of AEs resolved or were stable with no flares following discontinuation of study treatment and/or treatment with standard therapies for colitis.

### Malignancies

Based on the search using the SMQ of “malignant or unspecified tumors” and medical review, there were four (1 %) patients reported with events classified as malignant neoplasms, one each of: (a) breast cancer, diagnosed following treatment with placebo for 1 year and daclizumab 150 mg SC for 3 years and 2 months; (b) basal cell carcinoma, diagnosed following treatments with placebo for 1 year, daclizumab 300 mg SC for 1 year, and daclizumab 150 mg SC for 2 years and 9 months; (c) anal cancer, diagnosed following treatment with daclizumab 300 mg SC for 1 year, washout and re-initiation of daclizumab 300 mg SC for 1 year, and daclizumab 150 mg SC for approximately 5 months; and (d) pulmonary carcinoid tumor, diagnosed after treatment with placebo for 1 year, daclizumab 300 mg SC for 1 year, and daclizumab 150 mg SC for approximately 2 years. Of these cases of malignancy, anal cancer and pulmonary carcinoma were considered related to study treatment by the investigators. Overall, there was no observed pattern to the type of malignancies.

### Efficacy

**Fig. 3:**
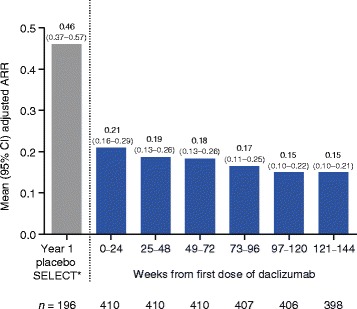
Adjusted annualized relapse rate (ARR) by 6-month intervals. Results from the SELECT placebo group have been published previously [3]. Adjusted ARR in SELECTED was estimated from a Poisson regression adjusted for the number of relapses in the year before study entry. Rates were estimated by time interval from the first dose of daclizumab received. *Gold et al. [3]

**Fig. 4:**
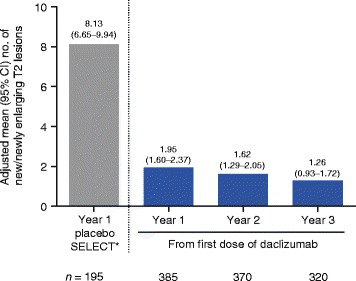
Adjusted mean number of new/newly enlarging T2 hyperintense lesions. Results from the SELECT placebo group have been published previously [3]. The adjusted mean number of T2 hyperintense lesions was estimated from a negative binomial regression adjusted for baseline number of T2 hyperintense lesions. *Gold et al. [3]

**Fig. 5:**
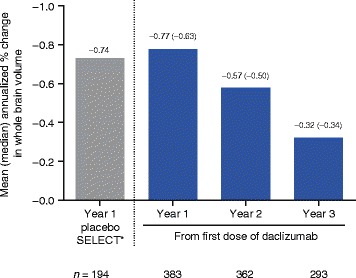
Mean (median) annualized percentage brain volume change (PBVC). Results from the SELECT placebo group have been published previously [3]. The annualized PBVC was determined with Structural Image Evaluation using Normalization of Atrophy (SIENA) and was calculated as percentage change divided by the number of days since the last scan multiplied by 365.25. For PBVC endpoints, patients with any post-baseline magnetic resonance imaging assessment in the efficacy population were included in the analysis. *Gold et al. [3]

The adjusted ARR analyzed at 6-month intervals from the first dose of daclizumab was 0.21 (95 % confidence interval (CI), 0.16–0.29) for weeks 0–24 and decreased to 0.15 (95 % CI, 0.10–0.21) by the weeks 121–144 interval (Fig. 3). The adjusted mean (95 % CI) number of new/newly enlarging T2 hyperintense lesions was 1.95 (1.60–2.37) in year 1 and decreased to 1.26 (0.93–1.72) by year 3 of treatment with daclizumab (Fig. 4). The mean (median) annualized PBVC was −0.77 % (−0.63 %) in year 1 and decreased to −0.32 % (−0.34 %) by year 3 of treatment with daclizumab (Fig. 5).

## Discussion

These interim findings from the SELECTED extension study indicate that although AEs continued to occur during the 3^rd^ year of patient treatment, there was no significant increase in the incidence of AEs and the safety profile remained consistent over time during extended treatment with daclizumab 150 mg SC in SELECTED, and that efficacy benefits were maintained for up to 3 years of treatment. The overall safety profile of daclizumab 150 mg SC in SELECTED was consistent with that observed in the previous studies, SELECT and SELECTION [3, 4] and in the 2- to 3-year, active-control phase 3 study of daclizumab 150 mg compared with interferon beta-1a [6]. In SELECT, infections, cutaneous AEs, and hepatic enzyme elevations were more common in daclizumab-treated patients than in placebo-treated patients [3], and the incidence of AEs did not increase in the second year of treatment with daclizumab while in SELECTION [4]. Similarly, 28 % in the safety population in this analysis reported cutaneous events compared with 13 % of patients in the placebo group in SELECT over a 52-week treatment period [3].

The yearly incidences of serious infections, cutaneous events, and liver enzyme abnormalities in this interim analysis of up to 3 additional years of daclizumab 150 mg SC treatment in SELECTED were similar to those observed in the first and second years of treatment in previous studies [3, 4], suggesting there was no apparent negative cumulative impact of long-term daclizumab treatment, especially with respect to hepatic function. Based on available data, there does not appear to be an increased rate of malignancy with extended daclizumab treatment; however, there is no comparator arm in this long-term follow-up. AEs associated with daclizumab treatment were generally self-limited or responsive to standard medical care. MS relapses categorized as SAEs were reported in 8 %, 6 % and 5 % of patients in Years 1, 2, and 3 of this study, respectively, compared with 21 % of patients treated with placebo during the treatment period of the SELECT study (unpublished observations, Biogen). One of the criteria used to define a SAE was any event requiring hospitalization or prolongation of hospitalization, and, thus, any such event was classified as an SAE. Adjusted ARR, the number of new/newly enlarging T2 hyperintense lesions, and the rate of brain volume loss remained low in year 3 of daclizumab 150 mg SC treatment, showing that the efficacy of daclizumab 150 mg SC is maintained for up to 3 years of treatment. Across the outcome measures evaluated, clinical and MRI MS disease activity in the third year of treatment with daclizumab 150 mg SC was consistently lower than that observed in the placebo group in year 1 in SELECT [3].

Limitations generally associated with long-term extension studies should be taken into consideration when interpreting the interim results of this study. Most importantly, SELECTED was an open-label study without a control group, similar to other open-label long-term extension studies [7, 8]. Secondly, despite the fact that there was a high re-enrollment rate from study to study in the SELECT TRILOGY [4] and a low rate of discontinuation due to AEs, patients non-responsive to treatment or those who are doing less well on treatment may have chosen not to continue to SELECTED, resulting in possible selection bias, favoring the retention of patients who either respond to or better tolerate daclizumab [4]. While this interim analysis may be affected by selection bias, any potential selection bias may be partially offset once the 6-year treatment period and 6-month postdosing safety follow-up for SELECTED are complete.

Previous patient exposure to the study drug was variable, ranging from 1–2 years treatment prior to SELECTED, either at the 300 mg or 150 mg dose, with or without a 24-week washout period at the beginning of year 2. This heterogeneity limits the strength of the efficacy conclusions; however, the results were consistent with analyses performed on the subset of SELECTED patients continuously treated with daclizumab 150 mg [9]. This variability may be more reflective of clinical practice rather than a typical clinical trial. Although conclusions regarding efficacy are limited by the lack of a control group, the effects of daclizumab on clinical and radiologic disease activity observed in year 1 were maintained for up to 3 years of treatment.

## Conclusions

The evaluation of long-term safety and efficacy of DMTs is important given the chronic nature of MS and the need for long-term treatment. Overall, the SELECTED findings provide evidence that risks associated with daclizumab were consistent with previous clinical trial experience with extended treatment, and reductions in MS disease activity on clinical and MRI outcomes were preserved over 3 years of treatment with daclizumab 150 mg SC. These findings suggest that daclizumab 150 mg SC may have a favorable benefit-risk profile for extended treatment in patients with RRMS. The SELECTED study is ongoing and the SELECT TRILOGY of clinical studies will provide data on up to 8 years of treatment, to inform the long-term safety and efficacy profile of daclizumab 150 mg SC monotherapy in patients with RRMS.

## Abbreviations

AE, adverse event; ALT, alanine transaminase; ARR, annualized relapse rate; AST, aspartate transaminase; CI, confidence interval; DMT, disease-modifying therapy; EDSS, Expanded Disability Status Scale; Gd^+^, gadolinium-enhancing; IL-2, interleukin 2; MedDRA, Medical Dictionary for Regulatory Activities; MRI, magnetic resonance imaging; MS, multiple sclerosis; PBVC, percentage brain volume change; RRMS, relapsing-remitting multiple sclerosis; SAE, serious adverse event; SC, subcutaneous; SD, standard deviation; SIENA, Structural Image Evaluation using Normalization of Atrophy; SMQ, Standardized Medical Dictionary for Regulatory Activities Query; SOC, System Organ Class; ULN, upper limit of normal

## Additional file

Additional file 1: Table S1. List of IECs and/or IRBs for SELECTED. (DOCX 20 kb)

## Footnotes

1 Daclizumab HYP, approved as Zinbryta™, has a different form and structure from an earlier form of daclizumab (Zenapax).
