# Supplementary material for: Safety and efficacy of daclizumab in relapsing-remitting multiple sclerosis: 3-year results from the SELECTED open-label extension study
Source: BMC Neurol. 2016 Jul 26;16:117. doi: 10.1186/s12883-016-0635-y (PMC4962457; doi:10.1186/s12883-016-0635-y)
Supplement: Additional file 1: Table S1. — List of IECs and/or IRBs for SELECTED. (DOCX 20 kb) [file 12883_2016_635_MOESM1_ESM.docx]

The Independent Ethics Commissions (IECs) and/or Institutional Review Boards (IRBs) that participated in this study are listed in Supplementary Table 1.

Supplementary Table 1: List of IECs and/or IRBs for SELECTED.

| Site Number | Principal Investigator | IEC/IRB Name and Address | Previous IEC/IRB Name and/or Address |
| --- | --- | --- | --- |
| **Czech Republic** | | | |
| 453 | Dr. Michal Dufek | Etická Komise Krajská nemocnice Liberec, Husova 10, Liberec, SEVEROCESKY KRAJ, 46063, Czech Republic  Etická komise, FN u sv. Anny v Brne, Pekarská 53, Brno, 656 91, Czech Republic * |  |
| 454 | Dr. Radomir Talab | Etická Komise Krajská nemocnice Liberec, Husova 10, Liberec, SEVEROCESKY KRAJ, 46063, Czech Republic  Etická komise Fakultní nemocnice Hradec Králové, Sokolská 581, Hradec Králové, Vychodocesky Kraj, 500 05, Czech Republic * | Etická komise Oblastní nemocnice Náchod, a.s. Purkyňova ul. 446, 547 01 Náchod * |
| 458 | Dr. Pavel Stourac | Etická Komise Krajská nemocnice Liberec, Husova 10, Liberec, SEVEROCESKY KRAJ, 46063, Czech Republic  Etická komise Fakultní nemocnice Brno, Jihlavská 20, Brno, JIHORMORAVSKY KRAJ, 625 00, Czech Republic * |  |
| 459 | Dr. Denisa Zimova | Etická Komise Krajská nemocnice Liberec, Husova 10, Liberec, SEVEROCESKY KRAJ, 46063, Czech Republic  Eticka komise Fakultni nemocnice Královské Vinohrady, Šrobárova 50, Praha 10, PRAHA, 100 34, Czech Republic * |  |
| 460 | Dr. Marta Vachova | Etická Komise Krajská nemocnice Liberec, Husova 10, Liberec, SEVEROCESKY KRAJ, 46063, Czech Republic  Nemocnice Teplice, Etická komise, Duchcovská 53, Teplice, SEVEROCESKY KRAJ, 415 29, Czech Republic * |  |
| **Germany** | | | |
| 353 | Dr. Björn Tackenberg | Ethik-Kommission der Medizinischen Fakultät der Friedrich-Alexander-Universität Erlangen-Nürnberg, Krankenhausstraße 12, Erlangen, BAYERN, 91054 Germany  Ethik-Kommission des Klinikums der Philipps Universität Marburg Fachbereich Medizin, Baldinger Strasse, Marburg, HESSEN, 35033, Germany * |  |
| 361 | Prof. Dr. Uwe Zettl | Ethik-Kommission der Medizinischen Fakultät der Friedrich-Alexander-Universität Erlangen-Nürnberg, Krankenhausstraße 12, Erlangen, BAYERN, 91054 Germany  Ethikkommission an der Medizinischen Fakultät der Universität Rostock, St.-Georg-Str. 108, Rostock, MECKLENBURG-VORPOMMERN, 18055 Germany * |  |
| 363 | Dr. Ralf Linker | Ethik-Kommission der Medizinischen Fakultät der Friedrich-Alexander-Universität Erlangen-Nürnberg, Krankenhausstraße 12, Erlangen, BAYERN, 91054 Germany |  |
| 365 | Prof. Dr. Patrick Oschmann | Ethik-Kommission der Medizinischen Fakultät der Friedrich-Alexander-Universität Erlangen-Nürnberg, Krankenhausstraße 12, Erlangen, BAYERN, 91054 Germany  Ethikkommission der Bayerischen Landesärztekammer, Mühlbaurstr. 16, München, BAYERN, 81677 Germany * |  |
| **Hungary** | | | |
| 550 | Dr. Attila Csányi | Egészségügyi Tudományos Tanács Klinikai Farmakológiai Etikai Bizottsága, Arany János utca 6-8, Budapest, H-1051, Hungary |  |
| 553 | Dr. Péter Diószeghy | Egészségügyi Tudományos Tanács Klinikai Farmakológiai Etikai Bizottsága, Arany János utca 6-8, Budapest, H-1051, Hungary |  |
| 555 | Dr. Krisztina Kovács | Egészségügyi Tudományos Tanács Klinikai Farmakológiai Etikai Bizottsága, Arany János utca 6-8, Budapest, H-1051, Hungary |  |
| 556 | Dr. Béla Clemens | Egészségügyi Tudományos Tanács Klinikai Farmakológiai Etikai Bizottsága, Arany János utca 6-8, Budapest, H-1051, Hungary |  |
| 558 | Dr. Etelka Jófejű | Egészségügyi Tudományos Tanács Klinikai Farmakológiai Etikai Bizottsága, Arany János utca 6-8, Budapest, H-1051, Hungary |  |
| 559 | Dr. Attila Valikovics | Egészségügyi Tudományos Tanács Klinikai Farmakológiai Etikai Bizottsága, Arany János utca 6-8, Budapest, H-1051, Hungary |  |
| 560 | Dr. Zita Jobbágy | Egészségügyi Tudományos Tanács Klinikai Farmakológiai Etikai Bizottsága, Arany János utca 6-8, Budapest, H-1051, Hungary |  |
| 561 | Prof. Dr. Dániel Bereczki | Egészségügyi Tudományos Tanács Klinikai Farmakológiai Etikai Bizottsága, Arany János utca 6-8, Budapest, H-1051, Hungary |  |
| 562 | Dr. Zsuzsanna Lohner | Egészségügyi Tudományos Tanács Klinikai Farmakológiai Etikai Bizottsága, Arany János utca 6-8, Budapest, H-1051, Hungary |  |
| 563 | Prof. Lászlo Csiba | Egészségügyi Tudományos Tanács Klinikai Farmakológiai Etikai Bizottsága, Arany János utca 6-8, Budapest, H-1051, Hungary |  |
| 564 | Dr. András Folyovich | Egészségügyi Tudományos Tanács Klinikai Farmakológiai Etikai Bizottsága, Arany János utca 6-8, Budapest, H-1051, Hungary |  |
| 565 | Dr. Péter Harcos | Egészségügyi Tudományos Tanács Klinikai Farmakológiai Etikai Bizottsága, Arany János utca 6-8, Budapest, H-1051, Hungary |  |
| 566 | Dr. Gabriella Kovács | Egészségügyi Tudományos Tanács Klinikai Farmakológiai Etikai Bizottsága, Arany János utca 6-8, Budapest, H-1051, Hungary |  |
| 567 | Dr. Mária Sátori | Egészségügyi Tudományos Tanács Klinikai Farmakológiai Etikai Bizottsága, Arany János utca 6-8, Budapest, H-1051, Hungary |  |
| **India** | | | |
| 100 | Dr. Raja Ram Agrawal | Swastik Ethics Committee, Laxmi Villa, B-15/5, Shiv Marg, Bani Park, Jaipur, RAJASTHAN, 302 016, India* | Institutional Ethics Committee, Fortis Escorts Hospital, Jawahar Lal Nehru Marg, Malviya Nagar, Jaipur, RAJASTHAN, 302 017, India * |
| 101 | Dr. Pahari Ghosh | Ethics Committee of Sri Auobindo Seva Kendra, 1-H, Gariahat Road (South), Jodhpur Park, Kolkata, WEST BENGAL, 700068, India * |  |
| 102 | Dr. Sangeeta Ravat | Institutional Ethics Committee, New UG/PG Hostel, Ground Floor, Seth GS Medical College & KEM Hospital, Parel, Mumbai, MAHARASHTRA, 400012, India * | Ethics Committee For Research On Human Subjects, Seth GS Medical College & KEM Hospital, Acharya Donde Marg, Parel, Mumbai, MAHARASHTRA, 400012, India * |
| 109 | Dr. Thomas Mathew | Institutional Ethics Committee, 1^st^ Floor, Zablocki Learning Center (St. John's Library), St. John's Medical College Hospital, Sarjapura Road, Bangalore, KARNATAKA, 560034, India * | Institutional Ethical Review Board, St. John's Medical College Hospital, Sarjapura Road, Koramangala, Bangalore, KARNATAKA, 560034 India * |
| 110 | Dr. A.K. Meena | Nizam's Institute of Medical Sciences Institutional Ethics Committee (NIEC), Punjagutta, Hyderabad, ANDHRA PRADESH, 500 082, India * |  |
| **Poland** | | | |
| 500 | Prof. Wieslaw Drozdowski | Komisja Bioetyczna przy Okregowej Izbie Lekarskiej w Lodzi, ulica Czerwona 3, Lódz, LODZKIE, 93-005, Poland |  |
| 501 | Dr. Waldemar Fryze | Komisja Bioetyczna przy Okregowej Izbie Lekarskiej w Lodzi, ulica Czerwona 3, Lódz, LODZKIE, 93-005, Poland |  |
| 502 | Dr. Jan Kochanowicz | Komisja Bioetyczna przy Okregowej Izbie Lekarskiej w Lodzi, ulica Czerwona 3, Lódz, LODZKIE, 93-005, Poland |  |
| 503 | Prof. Anna Kamińska | Komisja Bioetyczna przy Okregowej Izbie Lekarskiej w Lodzi, ulica Czerwona 3, Lódz, LODZKIE, 93-005, Poland |  |
| 505 | Prof. Krzysztof Selmaj | Komisja Bioetyczna przy Okregowej Izbie Lekarskiej w Lodzi, ulica Czerwona 3, Lódz, LODZKIE, 93-005, Poland |  |
| 506 | Prof. Andrzej Szczudlik | Komisja Bioetyczna przy Okregowej Izbie Lekarskiej w Lodzi, ulica Czerwona 3, Lódz, LODZKIE, 93-005, Poland |  |
| 508 | Prof. Anna Członkowska | Komisja Bioetyczna przy Okregowej Izbie Lekarskiej w Lodzi, ulica Czerwona 3, Lódz, LODZKIE, 93-005, Poland |  |
| 509 | Prof. Zbigniew Stelmasiak | Komisja Bioetyczna przy Okregowej Izbie Lekarskiej w Lodzi, ulica Czerwona 3, Lódz, LODZKIE, 93-005, Poland |  |
| 510 | Dr. Gabriela Kłodowska-Duda | Komisja Bioetyczna przy Okregowej Izbie Lekarskiej w Lodzi, ulica Czerwona 3, Lódz, LODZKIE, 93-005, Poland |  |
| 512 | Dr. Janusz Zbrojkiewicz | Komisja Bioetyczna przy Okregowej Izbie Lekarskiej w Lodzi, ulica Czerwona 3, Lódz, LODZKIE, 93-005, Poland |  |
| **Russia** | | | |
| 751 | Dr. Olga Vorobeva | Central Ethical Committee - Ethical Council at the Ministry of Health and Social Development of Russian Federation, 3, Rakhmanovsky Lane, Moscow, 127994, Russia  Local Ethical Committee of Non-State Healthcare Institution “N.A. Semashko Central Clinical Hospital #2 of OJSC ‘Russian Railways,’” 2, Budayskaya str., Moscow, 129128 Russia * |  |
| 752 | Dr. Leonid Zaslavsky | Central Ethical Committee - Ethical Council at the Ministry of Health and Social Development of Russian Federation, 3, Rakhmanovsky Lane, Moscow, 127994, Russia  Local Ethical Committee of State healthcare institution “Leningrad Regional Clinical Hospital,” 45-49, pr. Lunacharskogo, Saint Petersburg, 194291 Russia * |  |
| 753 | Dr. Zahira Mizieva | Central Ethical Committee - Ethical Council at the Ministry of Health and Social Development of Russian Federation, 3, Rakhmanovsky Lane, Moscow, 127994, Russia  Local Ethical Committee at Federal State Budgetary Institution “Federal Research and Clinical Centre for Specialized Medical Care and Technologies of the Federal Medical Biological Agency of Russia,” 28, Orekhovy bulvar, Moscow, 115682, Russia * |  |
| 758 | Dr. Irina Sokolova | Central Ethical Committee - Ethical Council at the Ministry of Health and Social Development of Russian Federation, 3, Rakhmanovsky Lane, Moscow, 127994, Russia  Local Ethics Committee of State Budgetary Healthcare Institution of Nizhny Novgorod region “City Hospital # 33 of Leninsky District of Nizhny Novgorod,” 54, Lenina prospect, Nizhny Novgorod, 603076, Russia * |  |
| 759 | Dr. Farit Khabirov | Central Ethical Committee - Ethical Council at the Ministry of Health and Social Development of Russian Federation, 3, Rakhmanovsky Lane, Moscow, 127994, Russia  Local Ethics Committee of State Independent Healthcare Institution “Republican Clinical Hospital of Rehabilitation Therapy of the Ministry of Health of the Republic of Tatarstan,” 13, Vatutina str., Kazan, 420021, Russia * |  |
| 761 | Dr. Natalia Nikolaevna Maslova | Central Ethical Committee - Ethical Council at the Ministry of Health and Social Development of Russian Federation, 3, Rakhmanovsky Lane, Moscow, 127994, Russia  Local Ethics Committee of Smolensk State Medical Academy (SSMA), 28, Krupskoy str., Smolensk, 214019 Based on Regional State Healthcare Institution “Smolensk Regional Clinical Hospital,” 27, Gagarina pr., Smolensk, 214018, Russia * |  |
| 763 | Dr. Irina Poverennova | Central Ethical Committee - Ethical Council at the Ministry of Health and Social Development of Russian Federation, 3, Rakhmanovsky Lane, Moscow, 127994, Russia  Local Ethics Committee of State Budgetary Healthcare Institution “Samara M.I. Kalinin Regional Clinical Hospital,” 159, Tashkentskaya str., Samara, 443095, Russia * |  |
| 764 | Dr. Nikolay Spirin | Central Ethical Committee - Ethical Council at the Ministry of Health and Social Development of Russian Federation, 3, Rakhmanovsky Lane, Moscow, 127994, Russia  Local Ethical Committee of State Institution of Healthcare of Yaroslavl region “Clinical Hospital # 8,” 39, Suzdalskoe shosse, Yaroslavl, 150030, Russia * |  |
| 765 | Dr. Nadezhda Malkova | Central Ethical Committee - Ethical Council at the Ministry of Health and Social Development of Russian Federation, 3, Rakhmanovsky Lane, Moscow, 127994, Russia  Local Ethics Committee of State Budget Healthcare Institution Novosibirsk Region “State Novosibirsk Regional Clinical Hospital,” 130, Nemirovicha-Danchenko str., Novosibirsk, 630087, Russia * |  |
| 766 | Dr. Semen Prokopenko | Central Ethical Committee - Ethical Council at the Ministry of Health and Social Development of Russian Federation, 3, Rakhmanovsky Lane, Moscow, 127994, Russia  Local Ethics Committee of State Budget Educational Institution of Higher Professional Education “Krasnoyarsk State Medical University Named After Prof. V.F. Voino-Yasenetsky of Ministry of Health Russia,” 1, Partizana Zheleznyaka str., Krasnoyarsk, 660022, Russia * |  |
| 767 | Dr. Alexey Rozhdestvensky | Central Ethical Committee - Ethical Council at the Ministry of Health and Social Development of Russian Federation, 3, Rakhmanovsky Lane, Moscow, 127994, Russia  Ethics Committee at State Budget Educational Institution of Higher Professional Education “Omsk State Medical Academy” of Ministry of Healthcare of Russia Federation based on Federal State Budget Institution of Healthcare “West Siberian Medical Centre of Federal Med Biological Agency,” Department of Neurology Postgraduate Education, 12, Lenina str., Omsk, 644043, Russia * |  |
| 768 | Dr Alexey Boiko | Central Ethical Committee - Ethical Council at the Ministry of Health and Social Development of Russian Federation, 3, Rakhmanovsky Lane, Moscow, 127994, Russia  Independent Ethics Committee at State Budgetary Healthcare Institution of the city of Moscow “Municipal Clinical Hospital # 24 of Department of Healthcare of the City of Moscow,, 6, Dvintsev ul., Moscow, 127018, Russia * |  |
| 769 | Dr Rim Magzhanov | Central Ethical Committee - Ethical Council at the Ministry of Health and Social Development of Russian Federation, 3, Rakhmanovsky Lane, Moscow, 127994, Russia  Expert Committee on Biomedical Ethics at State Budgetary Educational Institution of Higher Professional Education “Bashkir State Medical University” of Ministry of Health of Russian Federation, 3, Lenina Street, Ufa, 450000, Russia * |  |
| **Ukraine^1^** | | | |
| 900 | Dr. Nataliya Buchakchyys'ka | Central Ethics Commission Ministry of Health of Ukraine, Vulytsya Narodnogo opolcheniya, 5, Kyiv, Kiev, 03680 Ukraine  Local Ethics Committee at the Municipal institution “Zaporizhzhia Regional Clinical Hospital” of Zaporizhzhia Regional council, 10, Orekhovskoye shosse, Zaporizhzhia, 69600 Ukraine * |  |
| 901 | Dr. Nataliya Lytvynenko | Central Ethics Commission Ministry of Health of Ukraine, Vulytsya Narodnogo opolcheniya, 5, Kyiv, Kiev, 03680 Ukraine  Local Ethics Committee at Poltava Regional Clinical Hospital n.a. N.V. Sklifosovskogo, 23, Shevchenko Str., Poltava 36024, Ukraine * |  |
| 902 | Dr. Borys Palamar | Central Ethics Commission Ministry of Health of Ukraine, Vulytsya Narodnogo opolcheniya, 5, Kyiv, Kiev, 03680 Ukraine  Local Ethics Committee at the Kyiv City Clinical Hospital # 3, 26,Petra Zaporozhza Str., 02125, Kyiv, Ukraine * |  |
| 904 | Dr. Nataliya Voloshina | Central Ethics Commission Ministry of Health of Ukraine, Vulytsya Narodnogo opolcheniya, 5, Kyiv, Kiev, 03680 Ukraine  Local Ethics Committee at the State Institution “Institute of Neurology, Psychiatry and Narcology of National Academy of Medical Sciences of Ukraine,” 46,Acad. Pavlova Str., Kharkiv, 61068, Ukraine * |  |
| 906 | Dr. Larysa Sokolova | Central Ethics Commission Ministry of Health of Ukraine, Vulytsya Narodnogo opolcheniya, 5, Kyiv, Kiev, 03680 Ukraine  Local Ethics Commission of Kyiv City Clinical Hospital # 4, 17, Solomenskaya str., Kyiv, 03110 Ukraine * |  |
| 908 | Prof. Olexander Kozyolkin | Central Ethics Commission Ministry of Health of Ukraine, Vulytsya Narodnogo opolcheniya, 5, Kyiv, Kiev, 03680 Ukraine  Local Ethics Committee at the Municipal Institution “6th City Clinical Hospital,” 34, Stalevarov str., Zaporizhzhya, 69035, Ukraine * |  |
| 909 | Dr. Olena Moroz | Central Ethics Commission Ministry of Health of Ukraine, Vulytsya Narodnogo opolcheniya, 5, Kyiv, Kiev, 03680 Ukraine  Local Ethics Committee at the State Institution “Ukrainian State Research Institute of Medical and Social Problems Associated with Disability of Ministry of Health of Ukraine,” 1-A, Radyanskiy bst., Dnipropetrovsk, 49027, Ukraine * |  |
| 911 | Prof. Valeriy Pashkovskyy | Central Ethics Commission Ministry of Health of Ukraine, Vulytsya Narodnogo opolcheniya, 5, Kyiv, Kiev, 03680 Ukraine  Local Ethics Committee at the Municipal Medical Institution “Chernivtsi Regional Psychiatric Hospital,” 2, Musorhskyi str., Chernivtsi, 58018, Ukraine |  |
| 912 | Dr. Olena Statinova | Central Ethics Commission Ministry of Health of Ukraine, Vulytsya Narodnogo opolcheniya, 5, Kyiv, Kiev, 03680 Ukraine  Local Ethics Committee at the Municipal Medical Treatment Prophylaxis Institution “Donetsk Regional Clinical Territorial Medical Association,” 14, Prospect Ilicha, Donetsk, 83003, Ukraine * |  |
| 913 | Dr. Tetyana Kobys | Central Ethics Commission Ministry of Health of Ukraine, Vulytsya Narodnogo opolcheniya, 5, Kyiv, Kiev, 03680 Ukraine  Local Ethics Committee of Kyiv City Clinical Hospital # 4, 17, Solomenskaya str., 03110, Kyiv, Ukraine * |  |
| **United Kingdom** | | | |
| 301 | Dr. Clive Hawkins | NRES Committee North East-York, Room 002, TEDCO Business Centre, Viking Industrial Park, Rolling Mill Road, Jarrow, England NE32 3DT, UK | Previously called Northern and Yorkshire REC |
| 303 | Dr. Basil Sharrack | NRES Committee North East-York, Room 002, TEDCO Business Centre, Viking Industrial Park, Rolling Mill Road, Jarrow, England NE32 3DT, UK | Previously called Northern and Yorkshire REC |
| 304 | Dr. Cris Constantinescu | NRES Committee North East-York, Room 002, TEDCO Business Centre, Viking Industrial Park, Rolling Mill Road, Jarrow, England NE32 3DT, UK | Previously called Northern and Yorkshire REC |
| 306 | Prof. Jeremy Hobart | NRES Committee North East-York, Room 002, TEDCO Business Centre, Viking Industrial Park, Rolling Mill Road, Jarrow, England NE32 3DT, UK | Previously called Northern and Yorkshire REC |
| 312 | Dr. Eli Silber | NRES Committee North East-York, Room 002, TEDCO Business Centre, Viking Industrial Park, Rolling Mill Road, Jarrow, England NE32 3DT, UK | Previously called Northern and Yorkshire REC |

Note: For countries with both central and local ethics committees, the local ethics committee is marked with an asterisk (*).

^1^ The central ethics committee disbanded 12 April 2012.
